# The Cytoplasmic Location of Chicken Mx Is Not the Determining Factor for Its Lack of Antiviral Activity

**DOI:** 10.1371/journal.pone.0012151

**Published:** 2010-08-16

**Authors:** Camilla T. O. Benfield, Jon W. Lyall, Laurence S. Tiley

**Affiliations:** Department of Veterinary Medicine, University of Cambridge, Cambridge, United Kingdom; University of California San Francisco, United States of America

## Abstract

**Background:**

Chicken Mx belongs to the Mx family of interferon-induced dynamin-like GTPases, which in some species possess potent antiviral properties. Conflicting data exist for the antiviral capability of chicken Mx. Reports of anti-influenza activity of alleles encoding an Asn631 polymorphism have not been supported by subsequent studies. The normal cytoplasmic localisation of chicken Mx may influence its antiviral capacity. Here we report further studies to determine the antiviral potential of chicken Mx against Newcastle disease virus (NDV), an economically important cytoplasmic RNA virus of chickens, and Thogoto virus, an orthomyxovirus known to be exquisitely sensitive to the cytoplasmic MxA protein from humans. We also report the consequences of re-locating chicken Mx to the nucleus.

**Methodology/Principal Findings:**

Chicken Mx was tested in virus infection assays using NDV. Neither the Asn631 nor Ser631 Mx alleles (when transfected into 293T cells) showed inhibition of virus-directed gene expression when the cells were subsequently infected with NDV. Human MxA however did show significant inhibition of NDV-directed gene expression. Chicken Mx failed to inhibit a Thogoto virus (THOV) minireplicon system in which the cytoplasmic human MxA protein showed potent and specific inhibition. Relocalisation of chicken Mx to the nucleus was achieved by inserting the Simian Virus 40 large T antigen nuclear localisation sequence (SV40 NLS) at the N-terminus of chicken Mx. Nuclear re-localised chicken Mx did not inhibit influenza (A/PR/8/34) gene expression during virus infection in cell culture or influenza polymerase activity in A/PR/8/34 or A/Turkey/50-92/91 minireplicon systems.

**Conclusions/Significance:**

The chicken Mx protein (Asn631) lacks inhibitory effects against THOV and NDV, and is unable to suppress influenza replication when artificially re-localised to the cell nucleus. Thus, the natural cytoplasmic localisation of the chicken Mx protein does not account for its lack of antiviral activity.

## Introduction

Mx proteins are interferon (IFN)-induced dynamin-like GTPases found in all vertebrate species examined so far. The murine Mx1 protein was the first Mx protein to be discovered, when mice of the inbred A2G strain were found to resist doses of influenza A virus that were lethal to other mouse strains [1], [2]. Numerous studies have since confirmed the critical importance of Mx1 for influenza resistance in mice, independent from other IFN-induced genes [3], [4], [5], [6]. Mx proteins have been identified in diverse host species and exhibit a range of antiviral activities. While the murine Mx1 protein has specific activity against orthomyxoviruses, the human MxA protein inhibits a broad spectrum of viruses (including members of the *Orthomyxoviridae, Paramyxoviridae, Rhabdoviridae, Bunyaviridae, Hepadnaviridae* and *Asfaviridae*) [7]. Murine Mx1 and human MxA mediate their anti-influenza effects via distinct mechanisms: Mx1 is nuclear and inhibits primary transcription of the virus genome [8], while the cytoplasmic human MxA protein [9] affects an ill-defined post-transcriptional step [10] probably via an interaction with the nucleoprotein [11]. In the case of the related orthomyxovirus, THOV, human MxA has a particularly profound antiviral effect. Indeed, levels of MxA which are not sufficient to affect influenza growth are still inhibitory to THOV [12], and MxA is capable of reducing THOV titres by 1,000,000-fold in cell culture [12], while in similar experiments MxA reduced influenza titres by 200-fold [13]. In contrast, certain other Mx proteins lack demonstrable antiviral activities, such as duck Mx [14], the rat Mx3 protein [15] and the human MxB protein [13].

The chicken Mx protein was first cloned from a White Leghorn strain of chicken in 1995, and found to be devoid of detectable antiviral activity [16]. Subsequently, Ko et al. reported that the chicken Mx gene was highly polymorphic, and that the Mx alleles of some breeds of chicken did have activity against influenza virus and vesicular stomatitis virus (VSV) [17]. These authors showed that the amino acid at position 631 of the chicken Mx protein is a crucial determinant of anti-VSV activity (Asn631 is active and Ser631 is inactive against VSV) [18]. Although this finding sparked considerable interest in the prospect of selectively breeding chickens bearing Asn631 alleles for enhanced influenza resistance, subsequent investigations revealed that the Asn631 polymorphism did not confer influenza resistance in cell culture [19] or in chicken challenge studies [20]. Since the activity of the endogenous chicken Mx proteins might potentially be confounded by inadequate IFN-induced expression or the effect of other polymorphisms, we derived the Mx allele of the Japanese Shamo (SHK) breed of chicken by site-specific mutagenesis, since this was the only allele previously reported to inhibit influenza virus [17]. However, contrary to the previous report, our data showed that expression of the SHK chicken Mx protein did not inhibit influenza virus replication or gene expression [19].

The aim of this study was to evaluate whether the antiviral capacity of the chicken Mx protein was influenced by its cytoplasmic location. We therefore tested it against NDV, an economically important cytoplasmic RNA virus of chickens, and Thogoto virus, an orthomyxovirus known to be exquisitely sensitive to cytoplasmic Mx proteins. We also report the consequences of re-locating chicken Mx to the nucleus. Our analysis focused on the allele from the Shamo (SHK) chicken line (Asn631) which reportedly inhibited VSV and was previously regarded as the prototype functional chicken Mx allele [17]. In parallel, a Ser631 chicken Mx allele (8.1) was assessed (presumed non-functional since the Ser631 polymorphism destroys antiviral activity against VSV [18]), together with the known antiviral Mx proteins murine Mx1 and human MxA.

## Results

### Activity of Chicken Mx against Newcastle Disease Virus

NDV was chosen as a candidate to test for sensitivity to the chicken Mx protein because (i) NDV is an RNA virus belonging to the *Paramyxoviridae* family, several members of which are sensitive to the human MxA protein [21], [22], [23], [24], [25], [26]; (ii) NDV replicates exclusively in the cytoplasm of the host cell, where the chicken Mx protein is located and (iii) NDV is an important avian pathogen which poses a serious economic threat to the poultry industry.

To determine whether NDV was sensitive to inhibition by the chicken Mx protein, NDV-directed gene expression was measured using flow cytometry in 293T cells that were transiently transfected with Mx expression plasmids. The use of a recombinant GFP-expressing NDV strain (NDV-GFP) (described by Engel-Herbert et al. (2003) [27]) allowed the direct detection of virus-contingent gene expression by GFP autofluorescence. GFP autofluorescence in infected cells was previously shown to be as sensitive as viral antigen detection by immunofluorescence [27].

**Figure 1:**
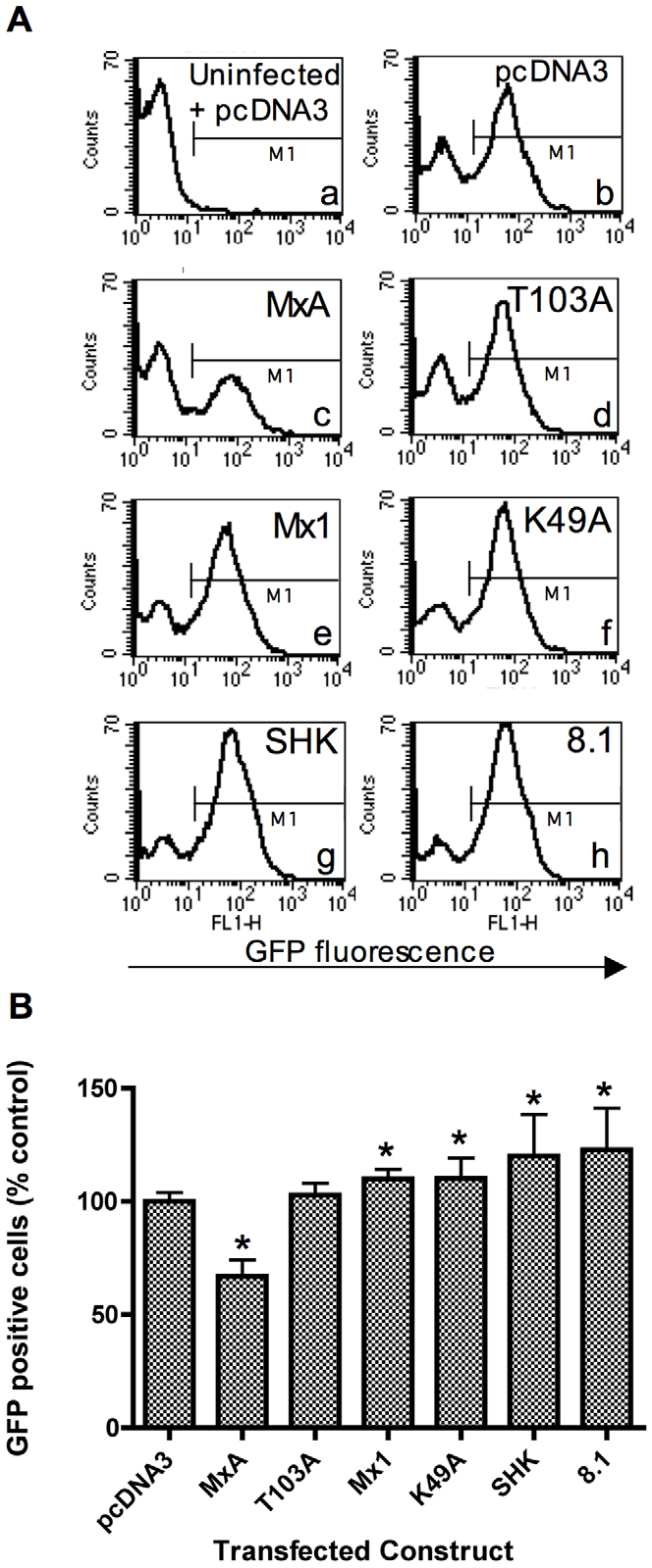
Chicken Mx proteins do not inhibit NDV-directed gene expression. 293T cells were co-transfected with a plasmid expressing the DsRed-express fluorescent protein and either pcDNA3 or a plasmid expressing the indicated Mx protein (human MxA, the MxA mutant T013A, murine Mx1, the Mx1 mutant K49A, wild type SHK (Asn631) or 8.1 (Ser631) chicken Mx). 48 h post-transfection, the cells were infected with NDV-GFP at an MOI which achieved approximately 60% infection (as determined by flow cytometry). 15 h post-infection, the cells were fixed and analysed by flow cytometry. Cells were then gated according to their expression of DsRed, and analysed for GFP fluorescence in the FL1-H channel. Panel A shows representative histograms for the GFP fluorescence in DsRed positive cells that were co-transfected with the indicated plasmids. Sub-panel (a) shows the background GFP fluorescence in uninfected, pcDNA3-transfected cells. The fluorescence threshold marker (M1) demarcates between GFP negative and positive cells. Panel B shows data derived from 6 replicates and bar heights show the % GFP positive cells expressed relative to that for the pcDNA3 control. The mean (and SD) are shown for DsRed positive cells co-transfected with the constructs as indicated. * indicates a significant difference (Students *t*-test) relative to pcDNA3 (p<0.05).

293T cells were co-transfected with an Mx expression construct and a plasmid expressing the DsRed-express fluorescent protein (Clontech) at a ratio of 3∶1. 48 h post-transfection, cells were infected with NDV-GFP. 15 h later the cells were analysed by flow cytometry. DsRed fluorescence was used to differentiate between the transfected and untransfected sub-populations and GFP fluorescence was used as a marker of productive infection by NDV. Fig. 1 shows representative frequency distributions of the GFP fluorescence in cells transfected with the indicated constructs (Panel A), and the mean % GFP positive cells from independent experiments (n = 6) (Panel B). To derive the data shown in Panel B, a fluorescence threshold marker (M1) was placed, as shown, to demarcate the infected GFP positive cells.

The background fluorescence detected in uninfected, pcDNA3-transfected cells is shown in sub-panel a (Fig. 1A). When pcDNA3 transfected cells were infected with NDV-GFP, two peaks were seen corresponding to uninfected and infected sub-populations of cells (Fig. 1A sub-panel b). In cells transfected with human MxA, there was a significantly lower proportion of GFP positive cells (67% relative to the pcDNA3 control) (p<0.01) (Fig. 1A sub-panel c and Fig. 1B). The mutant MxA protein, T103A, did not produce this reduction in the NDV-GFP signal (Fig. 1A sub-panel d and Fig. 1B). Both the SHK and 8.1 chicken Mx alleles also failed to inhibit NDV-GFP expression, and the proportion of GFP positive cells was actually slightly increased relative to pcDNA3-transfected cells (Fig. 1A sub-panels g and h and Fig. 1B). Similarly, cells transfected with murine Mx1 (or its K49A mutant) had a slightly higher proportion of GFP positive cells than cells transfected with pcDNA3 (Fig. 1A sub-panels e and f, and Fig. 1B).

### Activity of chicken Mx against Thogoto virus

The observation that NDV was sensitive to inhibition by human MxA but unaffected by murine Mx1 is consistent with the sub-cellular distribution of these two proteins and the cytoplasmic location of NDV replication. Chicken Mx also resides in the cytoplasm [16] but did not apparently inhibit NDV. However, the level of inhibition observed for human MxA was fairly modest, so we considered that the NDV assay was not a sufficiently rigorous test. In contrast, THOV is highly sensitive to human MxA [12], and we therefore tested whether THOV was sensitive to chicken Mx using a THOV minireplicon system. In minireplicon systems, a virus-like reporter RNA and the viral polymerase/NP replication complex proteins (3P/NP) are co-expressed from transfected plasmids. Transcriptionally active viral ribonucleoprotein complexes (vRNPs) are reconstituted intracellularly and the reporter RNA is transcribed and replicated, culminating in the expression of the reporter gene. Orthomyxovirus minireplicon systems have been widely used for the sensitive detection of the antiviral activity of co-expressed Mx proteins [11], [19], [28], [29].

**Figure 2:**
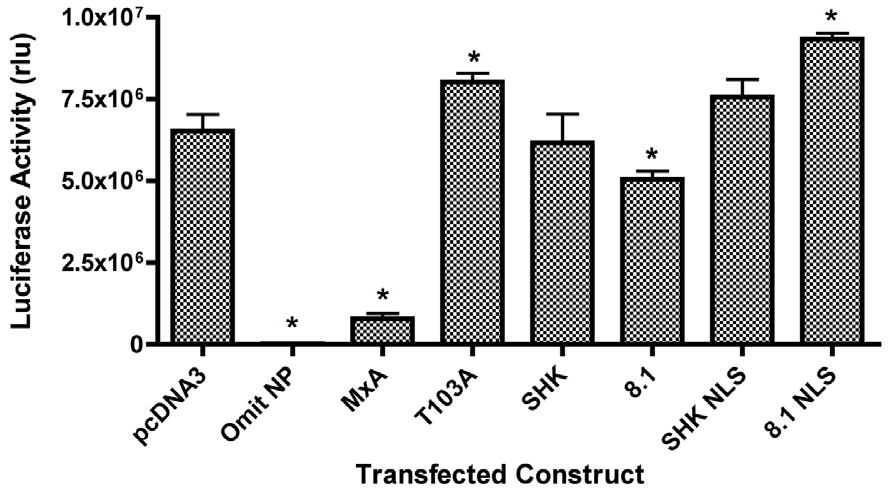
Chicken Mx proteins lack activity in a THOV minireplicon system. 293T cells were transfected with THOV PB1, PB2, PA and NP, a plasmid encoding a luciferase THOV minireplicon and a SEAP expressing plasmid, together with pcDNA3 or a plasmid expressing the indicated Mx protein (human MxA, the MxA T103A mutant, wild type chicken Mx proteins SHK (Asn631) and 8.1 (Ser631) and their nuclear-localised counterparts SHK NLS and 8.1 NLS). 48 h post-transfection, luciferase activity was measured and is shown as relative light units (rlu). The mean (and SD) of 3 replicates is shown. * indicates significant difference (Students *t*-test) relative to pcDNA3 (p<0.05).

Plasmids expressing THOV 3P/NP and a plasmid expressing a THOV-like negative sense luciferase RNA under the control of the human PolI promoter were transfected into 293T cells together with either Mx expression plasmids or empty vector (Fig. 2). Omission of the NP showed, as expected, that reporter activity was dependent on THOV vRNP reconstitution. Transfection of the human MxA expression plasmid caused a marked (∼90%) inhibition of luciferase activity in the THOV system, whereas a GTPase defective mutant MxA T103A was not inhibitory, thus demonstrating the sensitivity of this assay for detecting inhibition specific to a functional human MxA protein (Fig. 2). Transfection of the SHK chicken Mx expression plasmid did not alter luciferase activity compared to the pcDNA3 control. The 8.1 (Ser631) chicken Mx allele did produce a slight reduction (p<0.05) in reporter activity (relative to pcDNA3), although there was not a significant difference between the values obtained for the SHK and 8.1 alleles.

### Artificial nuclear re-localisation of chicken Mx proteins

Earlier work in our laboratory showed that chicken Mx failed to inhibit influenza gene expression or influenza minireplicon systems [19]. Other studies on the human MxA and rat Mx2 proteins showed that translocation of these proteins from their natural cytoplasmic location to the nucleus enhanced or revealed, respectively, anti-influenza activities [30], [31]. It was therefore hypothesised that artificial re-localisation of the chicken Mx protein from its usual cytoplasmic location to the cell nucleus, the site of orthomyxovirus transcription and replication, might confer antiviral activity on it.

**Figure 3:**
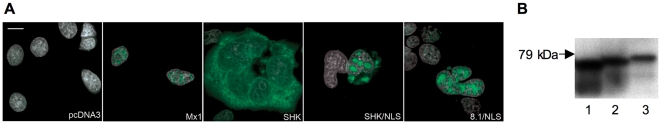
Nuclear re-localisation of chicken Mx. 293T cells were transfected with pcDNA3 or plasmids expressing murine Mx1, SHK chicken Mx, or chicken Mx constructs containing the SV40 NLS at their N-termini (SHK NLS and 8.1 NLS). Panel A: 48 h post transfection, cells were stained using a species cross-reactive Mx specific monoclonal antibody (M143) followed by a FITC-conjugated anti-mouse antibody. Nuclei were counterstained with DAPI. Merged images are shown composed of overlaid images from the FITC and DAPI channels. Scale bar measures 10 um. Panel B: 48 h post transfection, cell lysates were prepared and 3 ug of total protein were loaded per sample for SDS-PAGE (7.5% resolving, 4% stacking). Western blotting was performed the anti-Mx antibody M143, followed by an anti-mouse alkaline phosphatase-conjugated antibody and detection using ECL. Lane 1: murine Mx1; Lane 2: SHK chicken Mx; Lane 3: SHK NLS. The position of the 79 kDa size marker is indicated by the arrow.

In order to re-direct chicken Mx to the cell nucleus, the SV40 large T antigen nuclear localization signal (NLS) was introduced at the N-termini of the chicken Mx coding sequence of the SHK and 8.1 alleles (this being the same strategy used by previous investigators to re-localise human MxA [31]). Immunofluorescent staining of transfected 293T cells using a monoclonal antibody that cross-reacts with avian, human and mouse Mx proteins confirmed that the wild type SHK Mx protein was restricted to the cytoplasm whereas the NLS-tagged proteins were redirected to large, discrete structures of unknown identity within the cell nucleus (Fig. 3A). These structures were morphologically distinct from the small punctate bodies characteristic of murine Mx1 localisation (Fig. 3A and [32]). To check expression levels of the nuclear-localised chicken Mx constructs, Western blotting was performed with the anti-Mx monoclonal antibody. Murine Mx1 and chicken Mx proteins were detected consistent with their expected sizes (72 and 75 kDa respectively [16], [33]) (Fig. 3B lanes 1 and 2). Densitometry revealed that there was less than a 2-fold difference in the steady-state level of nuclear-localised SHK Mx (lane 3) relative to wild type SHK Mx (lane 2) and murine Mx1 (lane 1).

### Nuclear-localised chicken Mx does not inhibit influenza replication

**Figure 4:**
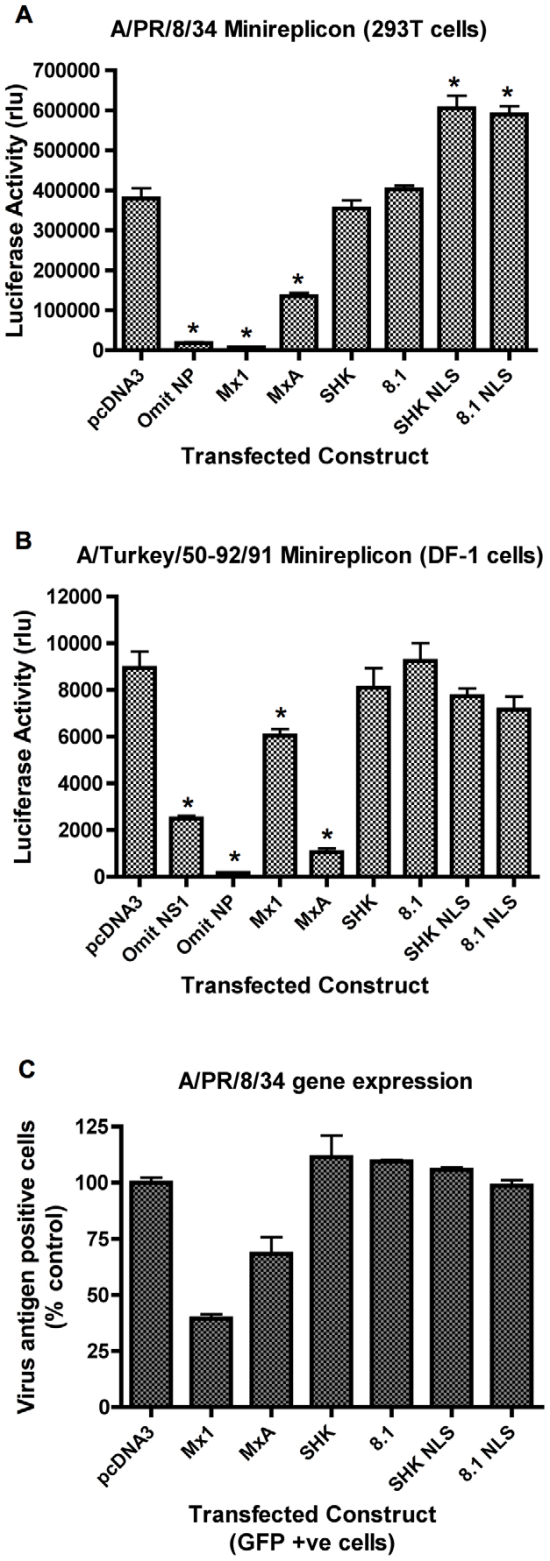
Nuclear localized chicken Mx lacks anti-influenza activity. Panel A: Effect on influenza A/PR/8/34 minireplicon system. 293T cells were co-transfected with plasmids expressing the PB1, PB2, PA and NP proteins of influenza A/PR/8/34, a plasmid encoding a luciferase minireplicon, and a SEAP-expressing plasmid, together with either pcDNA3 or a plasmid expressing the indicated Mx protein (murine Mx1, human MxA, wild type chicken Mx proteins SHK (Asn631) and 8.1 (Ser631) and their nuclear-localised counterparts SHK NLS and 8.1 NLS). 48 h post-transfection, luciferase activity was measured and is shown as SEAP-corrected relative light units (rlu). The mean (and SD) of 3 replicates is shown. * indicates p<0.05 (Students *t*-test) relative to pcDNA3. Panel B: Effect on influenza A/Turkey/England/50-92/91 minireplicon system. DF-1 cells were transfected with plasmids encoding A/Turkey/England/50-92/91 PB1, PB2, PA and NP, A/PR/8/34 NS1, an influenza minireplicon plasmid encoding luciferase, and a SEAP expressing plasmid together with pcDNA3 or a plasmid expressing the indicated Mx protein (murine Mx1, human MxA, wild type chicken Mx proteins SHK (Asn631) and 8.1 (Ser631) and their nuclear-localised counterparts SHK NLS and 8.1 NLS). 48 h post-transfection, luciferase activity was measured and is shown as relative light units (rlu). The mean (and SD) of 6 replicates is shown. * indicates p<0.05 (Students *t*-test) relative to pcDNA3. Panel C: Effect on influenza A/PR/8/34 gene expression. 293T cells were co-transfected with Mx-expressing plasmids (or pcDNA3) and pEGFP-C1 and infected after 48 h with influenza A/PR/8/34. 15 h post-infection, the cells were stained for influenza vRNP and analysed by flow cytometry. The percentage of antigen positive cells is expressed relative to that of the pcDNA3 control. The mean (and range) of 2 replicates is shown for GFP positive cells co-transfected with the indicated constructs.

The activity of the nuclear-localised chicken Mx was assessed using influenza minireplicon assays. In an A/PR/8/34 minireplicon system in 293T cells (Fig. 4A), co-transfection of murine Mx1 or human MxA resulted in significant reduction in luciferase reporter activity, while co-transfection of chicken Mx (SHK or 8.1) had no effect as anticipated from previous studies [19]. Both of the nuclear-localised chicken Mx alleles (SHK NLS and 8.1 NLS) paradoxically caused a small but reproducible increase in reporter levels in this assay (Fig. 4A). SV40 ori competition for T-Ag in the 293T cells is not the cause, as care was taken to maintain a constant ratio of origins in the various transfection cocktails. Next, the Mx constructs were tested in a minireplicon system based on the avian influenza strain A/Turkey/England/50-92/91 (H5N1) in chicken DF-1 cells, thus supplying chicken host cell factors that might be required for the function of chicken Mx. DF-1 cells are IFN competent [34] and plasmid transfection induces IFN in CEFs [35]. Therefore, the DF-1 cells were co-transfected with a plasmid expressing the A/PR/8/34 NS1 gene, which was previously shown to antagonise IFN induction in CEFs [35]. Omission of the NS1 plasmid from the transfection mix caused a significant decrease in luciferase levels consistent with partial suppression of the system by the induction of IFN (Fig. 4B). In this assay, the murine Mx1 and human MxA proteins were inhibitory, but the nuclear-localised chicken Mx proteins resembled their wild type counterparts in showing no inhibitory effect (Fig. 4B). Likewise, nuclear localised chicken Mx failed to inhibit reporter gene expression in the equivalent THOV minireplicon assay (Fig. 2).

Finally, the nuclear-targeted chicken Mx proteins were tested for their ability to inhibit influenza A/PR/8/34 gene expression, using the flow cytometry-based assay which was previously described [19]. Briefly, 293T cells were co-transfected with Mx expression plasmids and a plasmid expressing GFP in a ratio of 3∶1, and 48 h later infected with A/PR/8/34 at a multiplicity that achieved approximately 60% infection, as determined by FACS analysis using anti-influenza RNP-specific antibody, and phycoerythrin (PE)-conjugated secondary antibody. To identify the positively transfected sub-population, cells were gated for their GFP fluorescence and then analysed for PE staining in the FL-2 channel. As expected, transfection of either murine Mx1 or human MxA reduced the proportion of antigen positive cells compared to transfection with pcDNA3 (Fig. 4C). However, transfected 293T cells expressing SHK NLS or 8.1 NLS sustained equivalent levels of influenza (A/PR/8/34) gene expression compared to cells transfected with the empty vector pcDNA3 or with the wild type chicken Mx proteins (Fig. 4C).

## Discussion

There are several lines of evidence which indicate that sub-cellular localisation is key determinant of the antiviral activity of Mx proteins. Firstly, murine Mx1, which localises to the nucleus, is ineffective against cytoplasmic viruses whereas the cytoplasmic human MxA protein has a much broader spectrum of susceptible viruses including those that replicate in the cytoplasm. Secondly, artificial re-localisation of the human MxA protein to the nucleus was shown to enhance its ability to inhibit influenza virus [31] and influenza minireplicon systems [11]. Interestingly, nuclear-localised MxA acts like murine Mx1 and suppresses primary transcription [31]. Thirdly, the rat Mx2 protein has no anti-influenza activity when in its natural cytoplasmic location, but gains this ability upon translocation to the nucleus [30]. It was therefore reasonable to ascertain whether the sub-cellular distribution of chicken Mx was an important determinant in its antiviral activity (or lack thereof), and whether an otherwise inactive protein might gain antiviral activity upon nuclear re-localisation.

The avian paramyxovirus, NDV, was chosen as a representative cytoplasmic RNA virus relevant to chickens. Although we were able to demonstrate for the first time that NDV was susceptible to inhibition by wild type MxA, we were unable to detect any significant inhibition by chicken Mx (Fig. 1). The inability of murine Mx1 to inhibit NDV gene expression was expected since murine Mx1 is nuclear while all steps of the NDV life cycle occur in the cytoplasm. These data do not rule out an effect of chicken Mx on downstream events such as NDV packaging and egress, although there is no known precedent for Mx proteins targeting these events. The apparent small enhancement of NDV-GFP expression in cells transfected with either chicken Mx, murine Mx1 or the mutant K49A protein is of unknown biological relevance.

The THOV minireplicon system was highly sensitive to the human MxA protein, consistent with previous *in vitro* and *in vivo* reports which demonstrate that human MxA is particularly effective in restricting THOV [12], [36]. However, the prototype functional SHK chicken Mx allele (Asn631) did not have any inhibitory effect against the THOV minireplicon (Fig. 2). The minor but reproducible reduction in reporter levels associated with 8.1 chicken Mx (Ser631) may be indicative of some weak antiviral activity of this allele, but we are not convinced it is biologically significant, in view of the much greater reduction achieved by human MxA and the lack of a statistical difference between the values for the SHK and 8.1 alleles. These data demonstrate that the Asn 631 allele of chicken Mx does not inhibit the transcription/replication activity of the THOV polymerase.

The addition of the SV40 NLS at the N-terminus of the chicken Mx ORF successfully directed the protein to the nucleus (Fig. 3). However, nuclear-localised chicken Mx was still unable to inhibit influenza gene expression (Fig. 4C), influenza minireplicon systems conducted in both 293T and chicken DF-1 cells (Figs. 4A and 2B) or the THOV minireplicon (Fig. 2). The nuclear localised chicken Mx proteins unavoidably have additional N-terminal amino acids comprising the FLAG-tagged NLS epitope. N-termini of Mx proteins show considerable variability [16] and tolerate N-terminal alterations. We find that N-terminal FLAG tagged murine Mx1 retains its activity (data not shown), and others have reported similarly for N-terminal FLAG tagged MxA [37]. Therefore, while we cannot formally exclude the possibility, it is unlikely that the presence of the N-terminal FLAG tag/NLS accounts for the inability of nuclear localised chicken Mx to inhibit influenza. However, it should be noted that 15 out of a total of 18 positively selected codons are located in the N-terminal region of avian Mx proteins, suggesting some evolutionary significance for this region [38]. We therefore concluded that, subject to the above caveat, retargeting chicken Mx to the nucleus does not confer on it inhibitory properties against influenza virus.

Thus, in the range of antiviral assays described herein, chicken Mx lacks any antiviral capabilities, despite the presence of the Asn631 polymorphism. These data support other reports [19], [20] in demonstrating that the Asn631 polymorphism is not a decisive determinant of the antiviral activity of chicken Mx despite an earlier report to the contrary [18]. Recent evolutionary analyses show, using inter-specific comparisons of Mx sequences, that the codon at position 631 of the chicken Mx protein is not positively selected [38]. This also suggests that the Asn631 polymorphism is not associated with *in vivo* resistance to significant chicken pathogens (unless it is also associated with a deleterious phenotypic trait).

However, in view of the high level of polymorphism in the chicken Mx gene, it remains possible that alleles other than those tested here might have antiviral activity. Hence, the orthomyxovirus minireplicon assays and the flow cytometry-based assays used here could be used as efficient screens for antiviral alleles, which might then be harnessed to increase viral resistance via selective breeding. Other relevant viruses could also be tested (and in the case of VSV, retested) for their sensitivity to the Asn631 chicken Mx allele. In particular, infectious bursal disease virus, a chicken pathogen reported to be sensitive to human MxA [39], could be tested against chicken Mx in the future. However, it is interesting that the antivirally inactive human MxB protein affects nucleocytoplasmic trafficking and cell cycle progression [40], and that human MxA has effects upon cellular processes such as intracellular calcium signalling [41], cell motility [42], apoptosis [43], and endocytosis [44]. Therefore, despite the absence of an antiviral phenotype, it is clearly premature to consider chicken Mx as a functionless/vestigial protein, and timely to address its potential cellular effects.

## Materials and Methods

### Cells, viruses & plasmids

293T cells and DF-1 cells were obtained from the ATCC Cell Biology Collection and were grown according to ATCC guidelines. Influenza infections were performed with egg-grown A/PR/8/34 (Cambridge) (2×10^8^ PFU/ml) which was kindly provided by Dr Paul Digard (University of Cambridge). NDV-GFP is a recombinant lentogenic NDV strain (Clone-30) which contains the GFP gene inserted into the intergenic region between the fusion and hemagglutinin-neuraminidase proteins. This was previously characterised and kindly provided by Dr Angela Römer-Oberdörfer (Friedrich-Loeffler-Institutes, Insel Riems, Germany) [27]. Plasmids pcDNA-PB1, -PB2, –PA, –NP and –NS1 [45], [46], expressing the indicated proteins of A/PR/8/34 (H1N1) were kindly provided by Dr. Paul Digard. Plasmids PolI/II 50-92-PB1, -PB2, -PA and –NP express proteins from A/Turkey/England/50-92/91 (H5N1) and were generously provided by Prof. Wendy Barclay (Imperial College London) [47]. The construction of plasmids expressing an influenza virus-based luciferase minireplicon RNA under the control of either the human RNA polymerase I promoter or the chicken RNA polymerase I promoter was described previously [19]. Expression constructs for wild type and mutant murine Mx1 and human MxA proteins (pcDNA3-mMx1(wt), pcDNA3-mMx1(K49A), pcDNA3-HA-MxA(wt) and pcDNA3-HA-MxA(T103A)) were kindly provided by Dr Georg Kochs [48], [49]. MxA T103A [37] and Mx1 K49A [50] have single amino acid substitutions in their GTP binding domains that abolish GTPase and antiviral activity. The plasmids comprising the THOV minireplicon system were also kindly supplied by Dr Kochs. The pCAGGS expression constructs for THOV PB1, PB2, PA and NP were generated by insertion of the respective cDNAs from the T7-driven pBSK expression vectors [51] into pCAGGS-MCS under the control of the chicken β-actin promoter [52]. The luciferase-encoding THOV minireplicon was generated by exchange of the NP open reading frame of pHH21-vNP [51] for the firefly luciferase cDNA, yielding pHH21-vNP-FF-Luc. The SHK chicken Mx gene was derived by mutagenesis and the 8.1 chicken Mx gene was cloned from a commercial chicken line as previously described [19].

### Construction of nuclear localised Mx proteins

To introduce the SV40 T-Ag NLS [53] at the N-terminus of the chicken Mx sequences, a pair of annealed complementary oligonucleotides with overhangs compatible with NheI and AgeI were ligated into the parental plasmids (pchMxSHK and pchMx8.1 [19]) digested with NheI and AgeI. The resulting sequence encompassing these two sites (in bold) with codon triplets indicated was: 5′ **GCTAGC**GCTACAGGT ATG GAT TAC AAA GAC GAT GAC GAC AAG CCT AAG AAG AAG AGG AAG GTG GA**A CCG GT**A GAA CAG CAG AAC ATG 3′, which is predicted to encode the following peptide sequence with the indicated features in parentheses: MDYKDDDDK (FLAG tag) PKKKRKV (T-Ag NLS) EPVEQQN (vector derived stuffer) M (first amino acid of Mx ORF). The Mx coding region and flanking vector sequences for newly constructed plasmids were confirmed by sequencing.

### Plasmid transfection

Fugene 6 transfection reagent (Roche) was used to transfect DF-1 and 293T cells using a ratio of 3 µl Fugene 6: 1 µg DNA. The total mass of DNA transfected per well was 500 ng for a 24 well plate and 2 µg for a 6 well plate. The manufacturer's transfection protocol was followed.

### Immunofluorescence analysis of Mx expression

293T cells were seeded and transfected in glass chamber slides, which had been coated with poly-D-lysine (Sigma P6407). 48 h after transfection, cells were fixed for 10 min at room temperature with 2% formaldehyde in PBS and then permeabilised using 0.2% Triton X-100 with 10% goat serum in PBS. The monoclonal antibody M143 [54] was then added at a dilution of 1∶400 in PBS containing 0.1% Tween 20 and 10% goat serum. After incubation for 1 h, the slides were washed three times in PBS before incubation with a 1∶100 dilution of FITC conjugated anti mouse antibody (DakoCytomation F 0313) for 30 min in the dark. After a further three washes in PBS, the slides were mounted using Vectashield mountant and examined using a Leica DMRXA microscope using ×40 magnification. Images were captured using a Photometrics Sensys camera and Leica QFISH software (Leica Microsystems) in black and white through DAPI and FITC filter sets. The FITC and DAPI images were then given false colour before merging the images to give the final result. Magnification and contrast settings were kept constant for all images.

### Detection of Mx by Western Blotting

Cells were transfected in 24 well plates with equal amounts of plasmids expressing either murine Mx1, SHK Mx or SHK NLS. Cells were lysed 48 h post-transfection in chilled RIPA buffer (10 mM Tris pH 7.4, 1 mM EDTA, 150 mM NaCl, 1% w/v sodium deoxycholate, 1% v/v Triton-X100, 0.1% w/v SDS, 0.25 mM PMSF). Samples (3 µg total protein per lane) were subjected to discontinuous SDS-PAGE (7.5% resolving gel, 4% stacking gel) and then transferred onto a PVDF membrane using semi-dry blotting. The membrane was blocked in 1X blocking buffer (Sigma B 6429) for 1 h, and then incubated for 90 min with M143 (diluted 1: 500 in 1X blocking buffer). After washing, the membrane was then incubated for 60 min with an alkaline phosphatase-conjugated anti-mouse antibody (Sigma A-0162, diluted 1: 1000 in 1X blocking buffer). The CDP-*Star* Universal chemiluminescence detection kit (Sigma U-ALK) was then used in accordance with the manufacturer's instructions.

### Luciferase minireplicon reporter assays

For orthomyxovirus minireplicon assays in 293T cells, each well (24 well plate) received 25 ng of each of the plasmids expressing the viral PB1, PB2, PA and NP proteins, 100 ng of a human PolI-driven luciferase reporter plasmid, 50 ng of a plasmid expressing secreted alkaline phosphatase (SEAP) [55], and 250 ng of an Mx bearing plasmid or pcDNA3 control plasmid. For the A/Turkey/50-92/91 system in DF-1 cells, cells were transfected with 125 ng of a plasmid expressing the NS1 gene of A/PR/8/34, 25 ng of each of the plasmids expressing the PB1, PB2, PA and NP proteins, 100 ng of a chicken PolI-driven luciferase reporter plasmid, 50 ng of a plasmid expressing SEAP and 125 ng of an Mx bearing plasmid or pcDNA3 control plasmid. 48 h post-transfection luciferase expression was assayed using the Bright-Glo Luciferase Assay System (Promega) according to the manufacturer's instructions. SEAP activity was determined using the calorimetric assay described previously [55]. Where appropriate, luciferase activity was normalised to the SEAP activity for each well in order to account for minor differences in transfection efficiencies.

### Flow cytometric analysis of viral gene expression

293T cells were transfected with an Mx expressing plasmid (or pcDNA3) (1.5 ug) and a plasmid expressing a fluorescent protein (GFP or DsRed-express as appropriate) (0.5 ug). 48h post transfection, cells were infected with either A/PR8/34 or NDV-GFP. 15 h post-infection, the cells were trypsinised, washed in PBS and then fixed for 10 min at room temperature using 2% formaldehyde in PBS. For NDV-GFP infected cells, GFP autofluorescence was determined directly in fixed cells. Influenza (A/PR/8/34) gene expression was detected using indirect immunofluorescence. The cells were first permeabilised for 10 min (using 0.2% Triton X-100 with 10% goat serum in PBS), blocked for 15 min using 10% goat serum in PBS and then incubated at room temperature for 1 h with a 1∶200 dilution (in 0.02% Triton X-100 and 2% goat serum in PBS) of rabbit polyclonal antisera to FPV (Rostock) vRNP [56], kindly provided by Dr. Paul Digard (University of Cambridge). The cells were then washed three times in PBA (PBS, 0.1% BSA, 0.01% sodium azide) before incubation with a 1∶20 dilution of anti-rabbit R-phycoerythrin (PE) conjugated antibody (Sigma P-9537) on ice for 30 min in the dark. Finally, the cells were washed a further three times in PBA before detection of their fluorescence using a Becton Dickinson FACSCalibur. For each sample, either 2×10^4^ or 1×10^4^ cells were analysed for their GFP or viral antigen fluorescence (depending on the experiment). Cell Quest 3.3 software was used to analyse the data.

### Statistical Analysis

Data were analysed for statistical significance using a two-tailed Student's *t* test assuming populations of unequal variance. The number of independent data sets for each experiment is indicated in the figure legends. Where appropriate, and unless otherwise stated, data sets were compared to the pcDNA3 controls. The threshold for significance was p<0.05.

## References

[1] Lindenmann J (1962). Resistance of mice to mouse-adapted influenza A virus.. Virology.

[2] Lindenmann J (1964). Inheritance of Resistance to Influenza Virus in Mice.. Proc Soc Exp Biol Med.

[3] Arnheiter H, Skuntz S, Noteborn M, Chang S, Meier E (1990). Transgenic mice with intracellular immunity to influenza virus.. Cell.

[4] Grimm D, Staeheli P, Hufbauer M, Koerner I, Martinez-Sobrido L (2007). Replication fitness determines high virulence of influenza A virus in mice carrying functional Mx1 resistance gene.. Proc Natl Acad Sci U S A.

[5] Staeheli P, Haller O, Boll W, Lindenmann J, Weissmann C (1986). Mx protein: constitutive expression in 3T3 cells transformed with cloned Mx cDNA confers selective resistance to influenza virus.. Cell.

[6] Tumpey TM, Szretter KJ, Van Hoeven N, Katz JM, Kochs G (2007). The Mx1 gene protects mice against the pandemic 1918 and highly lethal human H5N1 influenza viruses.. J Virol.

[7] Haller O, Staeheli P, Kochs G (2009). Protective role of interferon-induced Mx GTPases against influenza viruses.. Rev Sci Tech.

[8] Krug RM, Shaw M, Broni B, Shapiro G, Haller O (1985). Inhibition of influenza viral mRNA synthesis in cells expressing the interferon-induced Mx gene product.. J Virol.

[9] Staeheli P, Haller O (1985). Interferon-induced human protein with homology to protein Mx of influenza virus-resistant mice.. Mol Cell Biol.

[10] Pavlovic J, Haller O, Staeheli P (1992). Human and mouse Mx proteins inhibit different steps of the influenza virus multiplication cycle.. J Virol.

[11] Turan K, Mibayashi M, Sugiyama K, Saito S, Numajiri A (2004). Nuclear MxA proteins form a complex with influenza virus NP and inhibit the transcription of the engineered influenza virus genome.. Nucleic Acids Res.

[12] Frese M, Kochs G, Meier-Dieter U, Siebler J, Haller O (1995). Human MxA protein inhibits tick-borne Thogoto virus but not Dhori virus.. J Virol.

[13] Pavlovic J, Zurcher T, Haller O, Staeheli P (1990). Resistance to influenza virus and vesicular stomatitis virus conferred by expression of human MxA protein.. J Virol.

[14] Bazzigher L, Schwarz A, Staeheli P (1993). No enhanced influenza virus resistance of murine and avian cells expressing cloned duck Mx protein.. Virology.

[15] Meier E, Kunz G, Haller O, Arnheiter H (1990). Activity of rat Mx proteins against a rhabdovirus.. J Virol.

[16] Bernasconi D, Schultz U, Staeheli P (1995). The interferon-induced Mx protein of chickens lacks antiviral activity.. J Interferon Cytokine Res.

[17] Ko JH, Jin HK, Asano A, Takada A, Ninomiya A (2002). Polymorphisms and the differential antiviral activity of the chicken Mx gene.. Genome Res.

[18] Ko JH, Takada A, Mitsuhashi T, Agui T, Watanabe T (2004). Native antiviral specificity of chicken Mx protein depends on amino acid variation at position 631.. Anim Genet.

[19] Benfield CT, Lyall JW, Kochs G, Tiley LS (2008). Asparagine 631 variants of the chicken Mx protein do not inhibit influenza virus replication in primary chicken embryo fibroblasts or in vitro surrogate assays.. J Virol.

[20] Sironi L, Williams JL, Moreno-Martin AM, Ramelli P, Stella A (2008). Susceptibility of different chicken lines to H7N1 highly pathogenic avian influenza virus and the role of Mx gene polymorphism coding amino acid position 631.. Virology.

[21] Carlos TS, Young D, Stertz S, Kochs G, Randall RE (2007). Interferon-induced inhibition of parainfluenza virus type 5; the roles of MxA, PKR and oligo A synthetase/RNase L.. Virology.

[22] Choudhary S, Gao J, Leaman DW, De BP (2001). Interferon action against human parainfluenza virus type 3: involvement of a novel antiviral pathway in the inhibition of transcription.. J Virol.

[23] Leroy MP, Baise EA, Pire GA, Desmecht DJ (2007). Contribution of MX dynamin, oligoadenylate synthetase, and protein kinase R to anti-paramyxovirus activity of type 1 interferons in vitro.. Am J Vet Res.

[24] Schneider-Schaulies S, Schneider-Schaulies J, Schuster A, Bayer M, Pavlovic J (1994). Cell type-specific MxA-mediated inhibition of measles virus transcription in human brain cells.. J Virol.

[25] Schnorr JJ, Schneider-Schaulies S, Simon-Jodicke A, Pavlovic J, Horisberger MA (1993). MxA-dependent inhibition of measles virus glycoprotein synthesis in a stably transfected human monocytic cell line.. J Virol.

[26] Zhao H, De BP, Das T, Banerjee AK (1996). Inhibition of human parainfluenza virus-3 replication by interferon and human MxA.. Virology.

[27] Engel-Herbert I, Werner O, Teifke JP, Mebatsion T, Mettenleiter TC (2003). Characterization of a recombinant Newcastle disease virus expressing the green fluorescent protein.. J Virol Methods.

[28] Dittmann J, Stertz S, Grimm D, Steel J, Garcia-Sastre A (2008). Influenza A virus strains differ in sensitivity to the antiviral action of the Mx-GTPase.. J Virol.

[29] Weber F, Haller O, Kochs G (2000). MxA GTPase blocks reporter gene expression of reconstituted Thogoto virus ribonucleoprotein complexes.. J Virol.

[30] Johannes L, Arnheiter H, Meier E (1993). Switch in antiviral specificity of a GTPase upon translocation from the cytoplasm to the nucleus.. J Virol.

[31] Zurcher T, Pavlovic J, Staeheli P (1992). Mechanism of human MxA protein action: variants with changed antiviral properties.. Embo J.

[32] Dreiding P, Staeheli P, Haller O (1985). Interferon-induced protein Mx accumulates in nuclei of mouse cells expressing resistance to influenza viruses.. Virology.

[33] Horisberger MA, Staeheli P, Haller O (1983). Interferon induces a unique protein in mouse cells bearing a gene for resistance to influenza virus.. Proc Natl Acad Sci U S A.

[34] Huang Z, Krishnamurthy S, Panda A, Samal SK (2003). Newcastle disease virus V protein is associated with viral pathogenesis and functions as an alpha interferon antagonist.. J Virol.

[35] Park MS, Shaw ML, Munoz-Jordan J, Cros JF, Nakaya T (2003). Newcastle disease virus (NDV)-based assay demonstrates interferon-antagonist activity for the NDV V protein and the Nipah virus V, W, and C proteins.. J Virol.

[36] Pavlovic J, Arzet HA, Hefti HP, Frese M, Rost D (1995). Enhanced virus resistance of transgenic mice expressing the human MxA protein.. J Virol.

[37] Ponten A, Sick C, Weeber M, Haller O, Kochs G (1997). Dominant-negative mutants of human MxA protein: domains in the carboxy-terminal moiety are important for oligomerization and antiviral activity.. J Virol.

[38] Berlin S, Qu L, Li X, Yang N, Ellegren H (2008). Positive diversifying selection in avian Mx genes.. Immunogenetics.

[39] Mundt E (2007). Human MxA protein confers resistance to double-stranded RNA viruses of two virus families.. J Gen Virol.

[40] King MC, Raposo G, Lemmon MA (2004). Inhibition of nuclear import and cell-cycle progression by mutated forms of the dynamin-like GTPase MxB.. Proc Natl Acad Sci U S A.

[41] Lussier MP, Cayouette S, Lepage PK, Bernier CL, Francoeur N (2005). MxA, a member of the dynamin superfamily, interacts with the ankyrin-like repeat domain of TRPC.. J Biol Chem.

[42] Mushinski JF, Nguyen P, Stevens LM, Khanna C, Lee S (2009). Inhibition of tumor cell motility by the interferon-inducible GTPase MxA.. J Biol Chem.

[43] Mibayashi M, Nakad K, Nagata K (2002). Promoted cell death of cells expressing human MxA by influenza virus infection.. Microbiol Immunol.

[44] Jatiani SS, Mittal R (2004). Expression of the antiviral protein MxA in cells transiently perturbs endocytosis.. Biochem Biophys Res Commun.

[45] Carrasco M, Amorim MJ, Digard P (2004). Lipid raft-dependent targeting of the influenza A virus nucleoprotein to the apical plasma membrane.. Traffic.

[46] Mullin AE, Dalton RM, Amorim MJ, Elton D, Digard P (2004). Increased amounts of the influenza virus nucleoprotein do not promote higher levels of viral genome replication.. J Gen Virol.

[47] Howard W, Hayman A, Lackenby A, Whiteley A, Londt B (2007). Development of a reverse genetics system enabling the rescue of recombinant avian influenza virus A/Turkey/England/50-92/91 (H5N1).. Avian Dis.

[48] Stertz S, Dittmann J, Blanco JC, Pletneva LM, Haller O (2007). The antiviral potential of interferon-induced cotton rat mx proteins against orthomyxovirus (influenza), rhabdovirus, and bunyavirus.. J Interferon Cytokine Res.

[49] Stertz S, Reichelt M, Krijnse-Locker J, Mackenzie J, Simpson JC (2006). Interferon-induced, antiviral human MxA protein localizes to a distinct subcompartment of the smooth endoplasmic reticulum.. J Interferon Cytokine Res.

[50] Pitossi F, Blank A, Schroder A, Schwarz A, Hussi P (1993). A functional GTP-binding motif is necessary for antiviral activity of Mx proteins.. J Virol.

[51] Wagner E, Engelhardt OG, Gruber S, Haller O, Kochs G (2001). Rescue of recombinant Thogoto virus from cloned cDNA.. J Virol.

[52] Niwa H, Yamamura K, Miyazaki J (1991). Efficient selection for high-expression transfectants with a novel eukaryotic vector.. Gene.

[53] Kalderon D, Roberts BL, Richardson WD, Smith AE (1984). A short amino acid sequence able to specify nuclear location.. Cell.

[54] Flohr F, Schneider-Schaulies S, Haller O, Kochs G (1999). The central interactive region of human MxA GTPase is involved in GTPase activation and interaction with viral target structures.. FEBS Lett.

[55] Berger J, Hauber J, Hauber R, Geiger R, Cullen BR (1988). Secreted placental alkaline phosphatase: a powerful new quantitative indicator of gene expression in eukaryotic cells.. Gene.

[56] Mahy BWJ, Carroll AR, Brownson JMT, McGeoch DJ (1977). Block to influenza virus replication in cells preirradiated with ultraviolet light.. Virology.

